# Multispectral, Fluorescent and Photoplethysmographic Imaging for Remote Skin Assessment

**DOI:** 10.3390/s17051165

**Published:** 2017-05-19

**Authors:** Janis Spigulis

**Affiliations:** Biophotonics Laboratory, Institute of Atomic Physics and Spectroscopy, University of Latvia, Riga, LV-1586, Latvia; janis.spigulis@lu.lv; Tel.: +371-2948-5347

**Keywords:** multispectral skin imaging, skin autofluorescence and photobleaching, photoplethysmography imaging

## Abstract

Optical tissue imaging has several advantages over the routine clinical imaging methods, including non-invasiveness (it does not change the structure of tissues), remote operation (it avoids infections) and the ability to quantify the tissue condition by means of specific image parameters. Dermatologists and other skin experts need compact (preferably pocket-size), self-sustaining and easy-to-use imaging devices. The operational principles and designs of ten portable in-vivo skin imaging prototypes developed at the Biophotonics Laboratory of Institute of Atomic Physics and Spectroscopy, University of Latvia during the recent five years are presented in this paper. Four groups of imaging devices are considered. Multi-spectral imagers offer possibilities for distant mapping of specific skin parameters, thus facilitating better diagnostics of skin malformations. Autofluorescence intensity and photobleaching rate imagers show a promising potential for skin tumor identification and margin delineation. Photoplethysmography video-imagers ensure remote detection of cutaneous blood pulsations and can provide real-time information on cardiovascular parameters and anesthesia efficiency. Multimodal skin imagers perform several of the abovementioned functions by taking a number of spectral and video images with the same image sensor. Design details of the developed prototypes and results of clinical tests illustrating their functionality are presented and discussed.

## 1. Introduction

Biomedical imaging has become a powerful tool for diagnostics and monitoring of human health condition. Apart from routine clinical imaging modalities (e.g., x-ray, ultrasound, endoscopy, computed tomography, magnetic resonance imaging), a number of advanced “open air” optical imaging methods and technologies have been introduced recently. Their main advantages are remote operation (avoids infection) and non-invasiveness (does not change the structure of tissues). Besides, digital imaging ensures quantitative documentation on the skin condition and its changes.

There are several commercially available skin diagnostic imaging devices for dermatologists (e.g., *SIAscope* [1], *MelaFind* [2], confocal microscopes [3], multi-photon tomographs [4]), but most of them are bulky, cable-connected to computers and also too expensive for GPs or small clinics. With a perspective on personalized medicine, new more compact and less expensive self-sustaining designs for skin imaging are preferable. The recently commercialized pocket-size digital dermatoscopes [5], video-microscopes [6] and smartphone-based solutions [7] have shown promising potential for primary skin diagnostics. Further developments of portable skin imaging technologies would facilitate their wider and more efficient implementation in hospitals and clinics. They may also prove useful for home monitoring of skin condition, follow-up after skin therapies and for some forensic applications, e.g., for age estimation of bruises [8]. 

This review paper (which follows a previous review [9]) presents the operational principles, designs and clinical test results of ten portable in-vivo skin imaging prototypes developed over the last five years at the Biophotonics Laboratory of the University of Latvia. Four groups of imaging devices are presented. Multi-spectral imagers offer possibilities for distant mapping of specific skin parameters (e.g., distribution of skin chromophore concentrations) so facilitating better diagnostics of skin malformations. Autofluorescence photobleaching rate imagers show a promising potential for skin tumor identification and margin delineation. Photoplethysmography video-imagers ensure remote detection of cutaneous blood pulsations and can provide real-time information on cardiovascular parameters and anesthesia efficiency. Finally, multimodal skin imagers perform several of the abovementioned functions by taking a number of spectral and video images with the same image sensor. All devices are portable and most of them wireless; original software solutions (not discussed here) provide fast data processing for obtaining clinically significant tissue parameters.

## 2. Materials and Methods

### 2.1. Prototype Devices for Multispectral Skin Imaging

Multispectral imaging is a method based on acquisition of a limited number (typically three to 10) of images within relatively narrow non-overlapping spectral bands—so-called spectral images [10]. The captured spectral images of skin can be further converted into parametric images, e.g., 2-D maps that specify distribution of skin chromophore concentrations [11,12]. In the visible spectral range, a relatively simple 3-chromophore skin model can be applied for obtaining chromophore distribution maps over the imaged area [13]. The hardware implementing this approach should ensure easy capture of three narrowband spectral images of skin as fast as possible. One option for that is subsequent narrowband illumination by means of different color LEDs and capturing one spectral image under each illumination mode [11]. This approach was examined earlier by a compact research grade RGB camera-LED illumination ring system [14]. As the next steps, three prototype devices comprising commercial consumer cameras and spectrally specific illuminators were developed.

#### 2.1.1. RGB-LED Add-On Illumination System for Smartphones

Thanks to the rapid development of communication technologies, a number of smartphone camera-based health assessment software applications are available, both in ambient light and under white LED illumination from the same phone [15,16]. Such applications may provide, for instance, information about the potential malignancy of skin lesions [7]. To extend the smartphone applications for skin evaluation, we developed a technique for mapping the main skin chromophores using a RGB light source specially designed as add-on for various smartphone models. 

**Figure 1:**
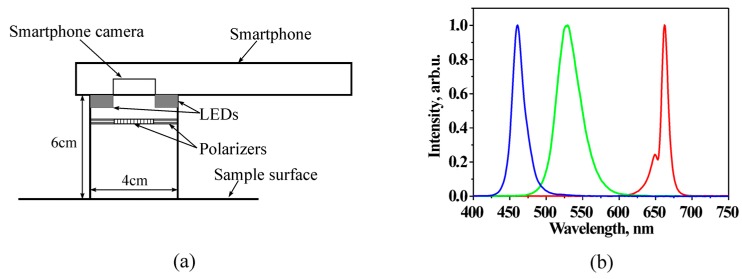
Design scheme of smartphone RGB illuminator (**a**) and normalized emission spectra of the used color LEDs (**b**) [18].

The system design scheme is presented at Figure 1. The smartphone is fixed on a flat sticky surface with a window for its rear camera [17], which is surrounded from bottom by a ring of LEDs mounted within cylindrical screening spacers (6 cm between the skin and camera). The ring includes four types of LEDs, with four diodes of each type: white—to find and adjust location of the skin malformation, and colored—with emission in blue (maximum at 460 nm), green (maximum at 535 nm), and red (maximum at 663 nm) spectral bands (Figure 1b) which are suitable for mapping of three skin chromophores. LEDs are operated in continuous mode and switched on and off manually or automatically by a special software using a Bluetooth connection between the smartphone and the illumination system. Illumination and image detection are performed normally to the skin surface. Two orthogonally oriented polarizers are used in front of the LEDs and smartphone camera, respectively, to reduce detection of skin specular reflection. Five AA 2800 mAh rechargeable battery blocks provide the system power supply. 

**Figure 2:**
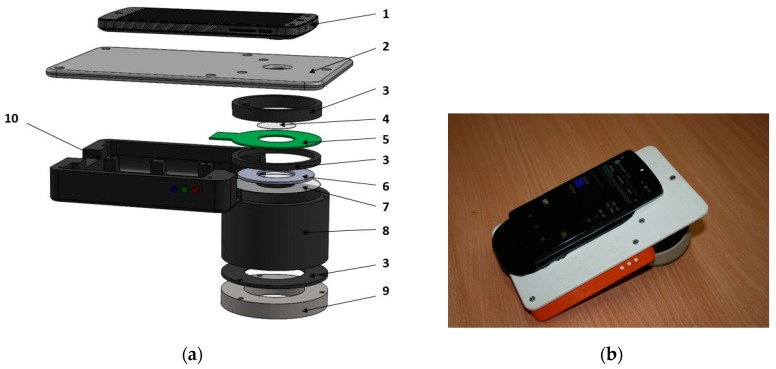
Design details of the smartphone-LED prototype device (**a**) and its outlook with a smartphone on it (**b**): 1—smartphone, 2—sticky fixing platform with a camera window, 3—holding ring, 4—polarizer of the detected light, 5—LED ring comprising four sets of LEDs, 6—light diffuser, 7—illumination polarizer (oriented orthogonally to the polarizer 4), 8—screening spacer, 9—silicone skin contact ring, 10—compartment for batteries and electronic components [17].

Figure 2a provides more design details of the prototype. A driver placed in the compartment 10 ensures Bluetooth wireless connection between the smartphone and illumination unit and enables automatic sequential on-off switching of the color LEDs within less than 1 s (one image for each illumination band) by command from the smartphone touchscreen. Specially developed software transmits the obtained spectral images via mobile network to a remote server that converts them into distribution maps of the three main skin chromophores (melanin, oxy- and deoxy-hemoglobin) and then transmits the maps back for displaying on the smartphone touchscreen. More details on the RGB-LED smartphone add-on prototype and its tests are provided in [17,18].

#### 2.1.2. Modified Multispectral Video-Microscope

A number of digital microscopes nowadays are small handheld devices connected to a PC via a USB cable; they can be applied also for visual skin assessment [19]. A typical digital microscope consists of a webcam with a high-powered macro-lens and a built-in LED light source. Advantages of such microscopes are their compactness, low power consumption and relatively low price, typically a few hundreds of USD. Most of the digital microscopes have white illumination source(s) and some of them–also ultraviolet illuminators [20]. These devices, however, cannot be used for detailed spectral analysis of skin. To overcome this drawback, we adapted a standard digital microscope (model *DinoLite* AD413, series AM-4013) for multispectral imaging by replacing the built-in LEDs with specifically selected color LEDs and by developing the LED management software. 

**Figure 3:**
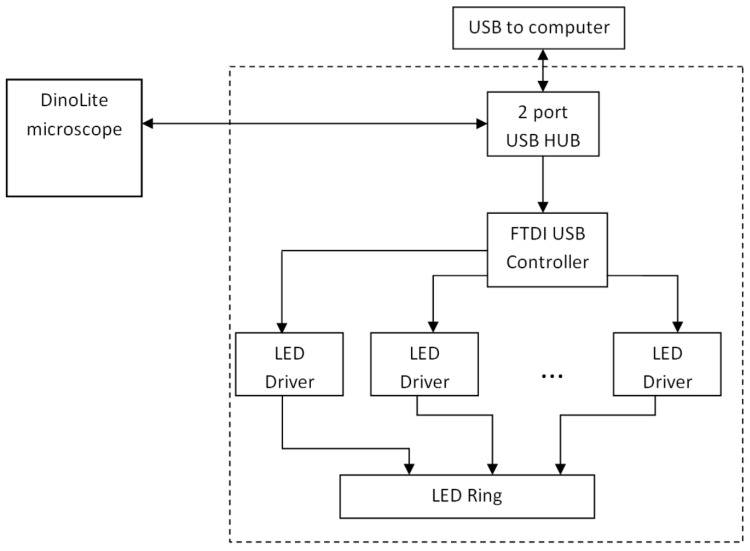
Block diagram of the modified video-microscope [21].

Figure 3 presents the block diagram of the custom-modified microscope. A standard white/UV LED illuminator ring has been removed and replaced by a lab-designed illuminator ring comprising sixteen LEDs combined in four groups: (1) four infrared 940 nm LEDs; (2) four red 660 nm LEDs; (3) four green 545 nm LEDs; and (4) four blue 450 nm LEDs. To each group of LEDs a current of 80 mA is fed by LED drivers that are controlled by a FTDI USB controller. The two-port USB hub provides control over the LEDs and the original CMOS image sensor of the microscope. The power support, LED switching and CMOS control are executed through the USB interface. 

**Figure 4:**
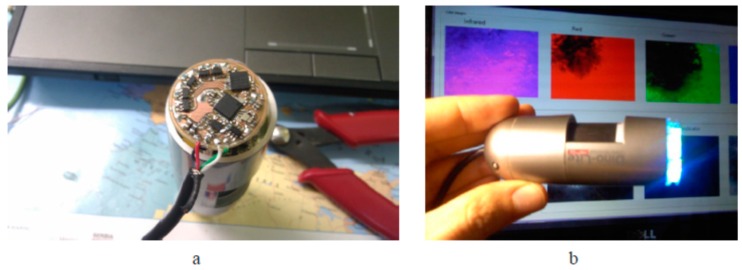
The developed LED control unit (**a**) and the modified video-microscope with replaced illumination unit (**b**) [21].

The custom-designed control unit module is installed on the rear side of microscope as shown on Figure 4a. The lab-made 16-LED ring is mounted on the front side (Figure 4b); it is even more compact than the original 8-LED *DinoLite* ring. For better homogeneity of skin illumination, a diffuser film is attached to the new LED-ring. To avoid detection of the directly reflected radiation from the skin surface (thus distinguishing the diffusely reflected radiation from the upper layers of skin), a pair of orthogonally-oriented polarizing filters are added: one of them directly after the diffuser, and the other-in front of the camera sensor matrix. The microscope is computer-controlled. The custom-developed program written in MatLab with a standard FTDI USB driver and a custom LED driver (written in C programming language) performs the control over LEDs and the acquisition of images. The software provides two image processing modes: (1) the preview mode, for focusing the microscope to the skin object, and (2) the video acquisition/processing mode, where the software performs sequential switching of LEDs and triggering of the video sensor. Each single measurement includes capturing of four frames (taken within the four wavelength bands). After recording, the images are stored in a 4-image matrix and saved as a data file for further processing. The image processing software allowed mapping of three above-mentioned skin chromophores, erythema index and the melanoma/nevus differentiation parameter [22]. More details on the modified video-microscope and its tests are available in [21,23,24].

#### 2.1.3. Prototypes for Smartphone Monochromatic Spectral Imaging of Skin at Multi-Laser Illumination

If skin is evenly illuminated by several laser sources, monochromatic spectral images can be extracted from a single RGB image file [25], thus making it possible to map several chromophores in a single snapshot. Such an approach speeds-up the image processing and excludes image artefacts caused by the tissue movements. First demonstration of skin hemoglobin snapshot RGB mapping under double-wavelength laser illumination was reported at [26]. Later mapping of three main skin chromophores under triple-wavelength laser illumination was demonstrated with laboratory [27] and smartphone-based [28] setups.

**Figure 5:**
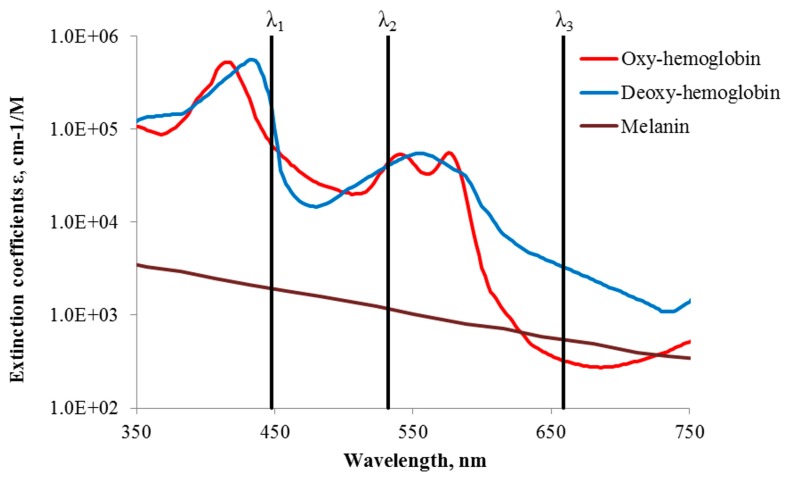
Absorption of three main skin chromophores [29,30] at three fixed wavelengths [28].

The general concept of snapshot skin chromophore mapping at fixed wavelengths is illustrated at Figure 5. Let us suppose that an RGB color image of skin is captured under illumination that comprises only three equal intensity spectral lines at wavelengths λ_1_, λ_2_ and λ_3_ (the vertical lines in Figure 5). With respect to the spectral sensitivity of RGB image sensor and the cross-talk between its detection bands at the fixed wavelengths, three monochromatic spectral images can be extracted from the color image data set by the technique described in [25,31]. If the skin surface reflection is suppressed (e.g., by means of two crossed polarizers), variations in chromophore composition induce changes of the diffusely reflected light intensities at each of the fixed wavelengths. Such variations in the pathology region relatively to the healthy skin can be estimated by measuring reflected light intensities from equally sized regions of interest in the pathology (*I_j_*) and the adjacent healthy skin (*I_oj_*). The ratios *I*_j_*/I*_jo_ at each pixel or pixel’s group of three monochromatic spectral images contain information on the concentration increase or decrease of all three regarded chromophores, which can be further mapped over the whole image area [28]. 

**Figure 6:**
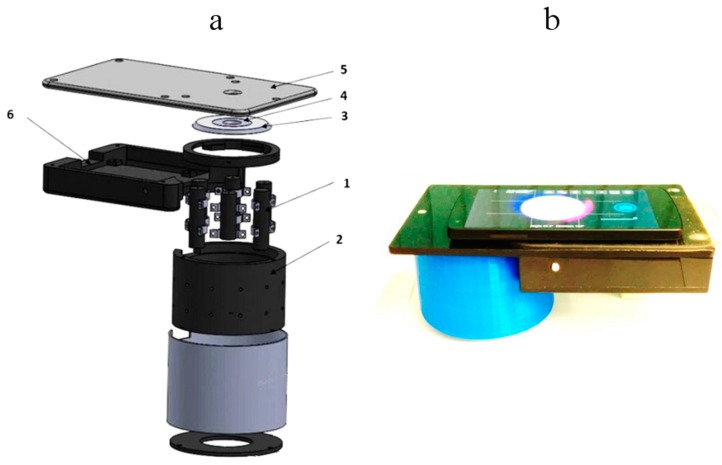
Design scheme of the 3-wavelength laser add-on illuminator (**a**) and the mobile prototype with smartphone on it (**b**): 1—laser modules (3 pairs, 448-532-659nm), 2—shielding cylinder, 3—collector of laser beams, 4—flat ring-shaped diffuser of laser light, 5—sticky platform for the smartphone, 6—electronics compartment [28].

A compact smartphone-compatible three wavelength illuminator has been designed, assembled and tested in the laboratory and clinics. Figure 6 shows the design details (left) and outlook of operating prototype with smartphone on it (right). Flat ring-shaped laser diffuser [17] ensures uniform three wavelength illumination of the round target area with diameter 40 mm. The illumination wavelengths 448 nm, 532 nm and 659 nm are emitted by three pairs of compact 20 mW power laser modules (models *PGL-DF-450nm-20mW-15011564, PGL-VI-1-532nm-20mW-15030443* and *PGL-DF-655nm-20mW-150302232* from Changchun New Industries Optoelectronics Tech. Co., Ltd., Changchun, China). Laser modules (1, Figure 6a—showing three out of six) of each equal-wavelength pair are mounted at opposite sides on the internal wall of a hollow 3D-printed plastic shielding cylinder 2; the round bottom opening of this cylinder is in contact with skin and forms the field of view for the smartphone camera, situated 80 mm apart. All six coaxial laser beams are pointed to the 45-degree conical reflecting edge of a Plexiglass transparent disc 3 (beam collector); after reflections they are turned radial towards the internal ring-shaped flat milky-Plexiglas diffuser 4. In result, the flat diffuser 4 evenly illuminates the 65 mm distant skin target area simultaneously by the three laser wavelengths.

The smartphone is placed on flat sticky platform 5 (Figure 6a) with a round window for the smartphone rear camera, co-aligned with the internal opening of the diffuser 4. The round camera window is covered by a film polarizer; another film with orthogonal direction of polarization covers the diffuser 4 from bottom, so avoiding detection of skin surface-reflected light by the smartphone camera. We used a model *Google Nexus5* smartphone comprising an 8Mpx *SONY IMX179* image sensor with known RGB-sensitivities; spatial resolution of the imaging system was better than 0.1 mm. 

**Figure 7:**
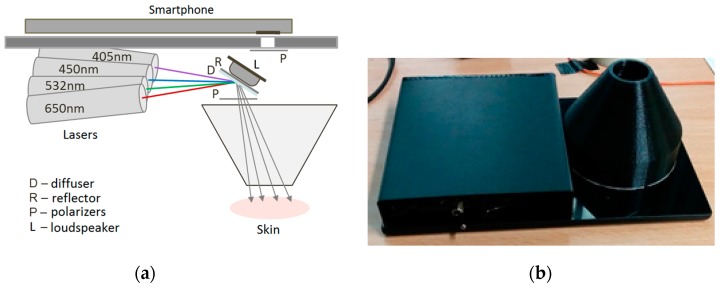
Design scheme (**a**) and outlook (**b**) of the prototype device for switchable 4-wavelengths skin illumination [33,34].

The single-snapshot RGB technique is not applicable for express-mapping of more than three skin chromophores. The double-snapshot approach [32] for obtaining four monochromatic images has been implemented in a model device comprising switchable four laser illuminator and a smartphone. Figure 7 shows the design scheme and outlook of a smartphone add-on illuminator intended for mapping of four skin chromophores, e.g., melanin, oxy-hemoglobin, deoxy-hemoglobin and bilirubin. Two of the laser modules can be manually switched on and off, so providing two sets of 3-wavelengths illumination (405, 532, 650 nm and 450, 532, 650 nm). Four rechargeable AA-type batteries are used for power supply. Relatively uniform illumination of round skin spot (dia. 18 mm) is provided by an advanced optical design which also reduces laser speckle artefacts [33]. More details on the multi-laser smartphone add-on prototypes and their test results can be found in [17,28,34].

### 2.2. Prototype Device for Skin Fluorescence Imaging With a Smartphone

**Figure 8:**
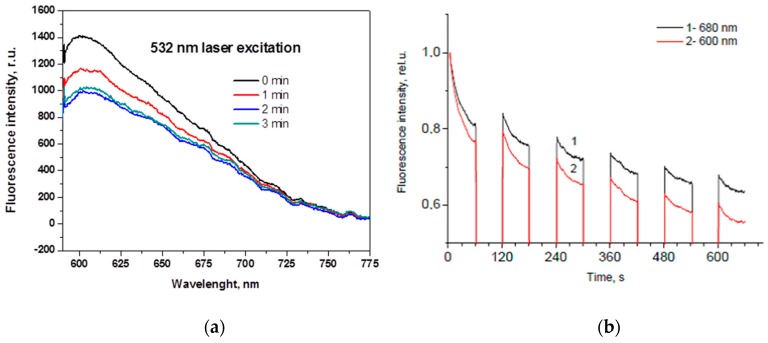
Skin autofluorescence photobleaching at continuous 532nm laser irradiation (~85 mW/cm^2^): (**a**)—temporal changes of the emission spectrum [36]; (**b**)—partial recovery of the autofluorescence intensity at two wavelengths after interrupted excitation [37].

If the light is absorbed in skin, it can be further re-emitted at longer wavelengths as autofluorescence (AF), i.e., self-fluorescence without any specific additives on or inside the skin. There is a number of fluorescing compounds called fluorophores in the upper skin, each with its specific emission spectrum. Even if excited by a narrow laser line, several emission spectra overlap and skin autofluorescence spectrum usually is bell-shaped, without a pronounced structure. Besides, a phenomenon called autofluorescence photobleaching (AFPB) normally takes place: the in-vivo skin emitted intensity decreases during continuous optical excitation and does not fully recover after interruptions of the excitation (Figure 8). AFPB causes some interesting effects like low power radiation induced “fingerprints” on in-vivo skin [35].

The decrease of skin autofluorescence intensity during the laser exposure in most cases can be approximated by a double-exponential expression:

*I*(*t*) = *a* exp(−*t*/τ_1_) + *b* exp(−*t*/τ_2_) + *A* (1)

where *I*—autofluorescence intensity at a fixed wavelength band, *t*—time, *a* and *b*—weighting coefficients, *A*—the “bottomline” constant, and τ_1_, τ_2_—the “fast” and “slow” AFPB rate coefficients, respectively. The “fast” AFPB (when sharp intensity decrease is observed) usually takes place during the first 5–15 s of the irradiation, while the “slow” AFPB continues up to several min.

Our first set-up for skin AFPB imaging comprised laser illuminator and consumer photo camera equipped with a band-pass filter in front of the objective; it was operating at a slow video-mode (~2 frames/s) [38]. After the image processing, distribution of τ-values over the imaged skin area was mapped and analyzed. This study showed that the τ-values are sensitive to skin structural changes—e.g., AFPB rates detected from melanin-pigmented nevi were always slower than those detected from healthy skin. Thus, the AFPB rate measurements and spatial mapping may have a potential for skin diagnostics and recovery monitoring, as well as for better skin tumor margin delineation. 

**Figure 9:**
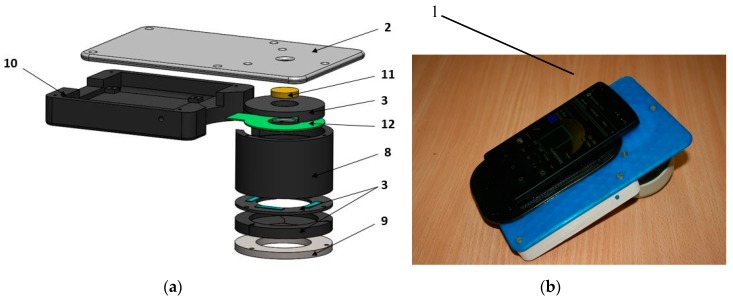
Design scheme (**a**) and outlook (**b**) of the prototype device for skin fluorescence imaging with a smartphone: 1—smartphone, 2—sticky fixing plate with camera window, 3—mounting rings, 8—cylindrical screening spacer, 9—silicone skin-contact ring, 10—battery/electronics compartment, 11—long-pass filter, 12—LED ring [17].

As the next step, a smartphone-compatible technique for acquisition and analysis of violet LED excited skin fluorescence intensity and AFPB rate distribution images has been developed and clinically tested [39]. Design of the prototype device is illustrated on Figure 9. For parametric mapping of skin AF intensity decrease rates, a sequence of AF images under continuous 405 nm LED (model LED Engin LZ1-00UA00-U8, spectral band half-width 30 nm) excitation at a power density of ~20 mW/cm^2^ with framerate 0.5 fr/s is recorded for 20 s. Four battery-powered violet LEDs placed within a cylindrical light-shielding wall (which also ensures a fixed 60 mm distance between the smartphone camera and skin) evenly irradiate a 40 mm round spot of the examined skin tissue. A long pass filter (>515 nm) is placed in front of the smartphone camera to prevent detection of the exciting LED emission. The battery/electronics compartment comprises a set of rechargeable batteries and a Bluetooth Low Energy (BLE) module with a driver for communications between the smartphone and illumination unit. 

The recorded RGB fluorescence images were further separated to exploit the R- and G-images for imaging of skin autofluorescence in the red and green spectral bands, respectively. Due to spectral cut-off by the 515 nm long pass filter, the B band images served only for reference. A *Samsung Galaxy Note 3* smartphone comprising integrated CMOS RGB image sensor with resolution of 13 MP was used for image acquisition. All images were taken at the following settings: ISO-100, white balance—daylight, focus—manual, exposure time—fixed 200 ms. More details on the skin fluorescence imager design and its test results are provided in [17,39].

### 2.3. Photoplethysmography Video-Imaging Prototypes

The incident cw light can be reflected from the skin surface and also may enter its epidermal and dermal layers where light can be absorbed and/or scattered. A part of back-scattered photons have been travelled via the skin dermal layer where arterial blood volume periodically changes with each heartbeat. As a consequence, also total blood absorption changes with each heartbeat and the back-scattered light intensity is modulated—the detected so-called remission photo- plethysmography (PPG) signal comprises a relatively stable DC component, determined by absorption of the “static” skin structures, and a pulsatile AC component caused by the periodically changing blood absorption [40]. The pulsatile remission PPG signals can be detected not only by specially designed skin contact probes [41], but also distantly, e.g., by video-imaging of skin with subsequent signal processing [42]. This technique is called PPG-imaging (PPGI) or remote PPG (rPPG). The captured video-signals consist of a number of image frames taken at a definite frame rate, e.g., 20 frames per second. Consequently, during one heart activity cycle (~1 s) 20 skin images are obtained, each at different phase of the sub-cutaneous pulse wave. If consecutive frames are compared, the skin-remitted light intensity detected from a fixed area increases and decreases with time, forming the periodic PPGI signal. Specific software [43] allows extracting the arterial pulsations from the video-signal over the whole imaged skin area. The amplitude of PPG peaks may differ between the image pixels due to different blood perfusion of the skin tissues—especially if there is a burn or other dermal skin damage. After the image processing, parametric maps of the PPG signal amplitude distribution (or blood perfusion maps) can be constructed [44]. 

#### 2.3.1. PPGI Prototype for Palm Anesthesia Monitoring

**Figure 10:**
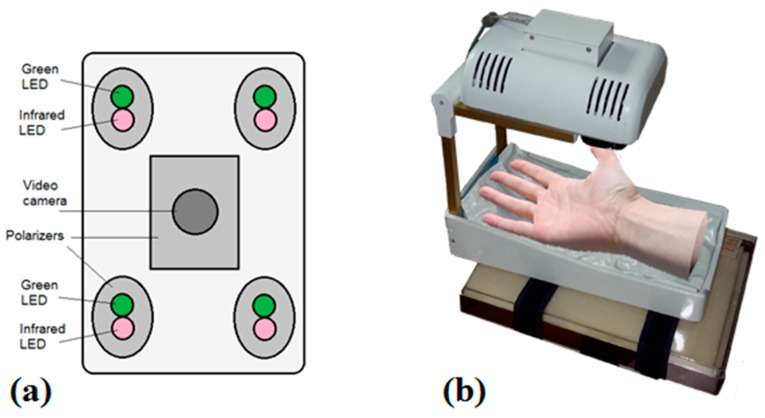
Dual wavelength photoplethysmography imaging device: bottom view of the imaging system-camera and light sources (**a**) and the whole device with vacuum pillow supporting the palm (**b**) [46].

Figure 10 illustrates the recently designed PPGI prototype device for distant monitoring of anesthesia efficiency during the palm surgeries. The system was intended for recording signals from the curved surface of the hand (dorsal or ventral aspect). The illuminator comprised four bispectral light sources, each consisting of two high-power LED emitters (Roithner LaserTechnik GmbH, Vienna, Austria; green: λ = 530 nm, 3 W and infrared: λ = 810 nm, 1 W). To achieve uniform illumination of skin surface, adjustable LED intensity control was introduced via PC based custom developed software. The two wavelengths of illumination were chosen in order to control blood pulsations at two different vascular depths in real time [45]. 

The microcontroller board (Arduino Nano, Arduino, New York, NY, USA) provided sequential switching of green and IR LEDs and triggering of the captured video frames. The camera control was performed by *uEye* software using manual trigger mode, fixed exposure time, 2 × 2 pixels binning and triggered at 60 frames/s. The monochromatic camera (8 bit CMOS *IDS-uEye UI-1221LE*) was equipped with an *S-mount* 1/2 inch F = 4 mm low distortion, wide-field lens (Lensagon, Lensation GmbH, Karlsruhe, Germany). The camera lens was placed at 15 cm distance from the skin surface so ensuring full view of adult palm (20 cm × 15 cm field of view). In order to reduce skin specular reflectance, orthogonally oriented polarizers were placed behind the camera and all four light sources, respectively. The plastic parts were produced by a 3D printer (*Prusa i3*, custom made, Latvia). The device comprised a plastic enclosure filled with an adjustable vacuum pillow (40 × 20 AB Germa, Kristianstad, Sweden) as the palm support.

#### 2.3.2. Universal Compact PPPGI Prototypes

**Figure 11:**
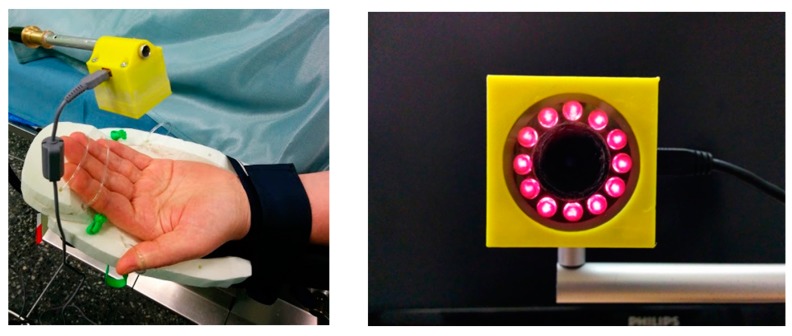
The compact PPGI prototype device in operation (**left**) and the front view of the device (**right**).

Another, much smaller PPGI prototype device (Figure 11) for more universal applications related to skin microcirculation control has been developed, as well. It involves near-infrared LED illuminator and a video camera, both placed in custom designed 3D-printed case (4 cm × 4 cm × 4 cm). The illuminator comprises a ring of twelve circularly oriented near-infrared LEDs (peak wavelength 760 nm, current 20 mA each), connected in parallel; stabilized illumination is provided by LED driver (cat4104). Video acquisition is performed by a board-level CMOS video camera (*IDS uEye UI-1221LE*), resolution 752 × 480 pixels, maximum framerate 87 fps., 8-bit monochrome). The camera is equipped with low-distortion S-mount 4 mm lens (Lensagon). In order to reduce skin specular reflectance, cross-oriented polarizers are placed behind the light sources and in front of the camera, respectively. The infrared cut-off filter KC-15 (>700 nm) also is placed in the front of the camera, in order to minimize the influence of ambient illumination. USB-2 cable connection to PC ensures both video-signal processing and the power supply. 

**Figure 12:**
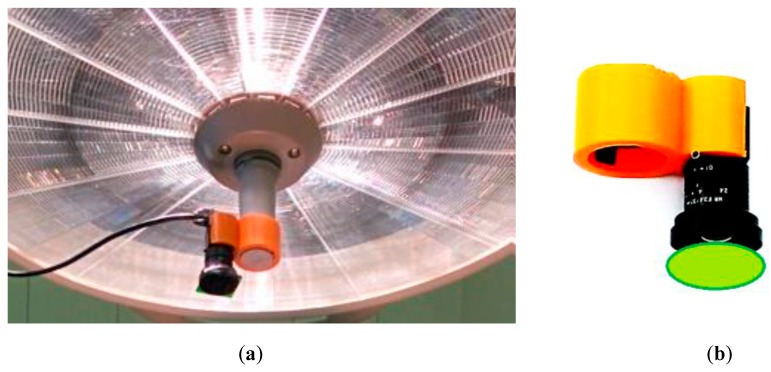
The PPGI device attached to a surgical lamp (**a**) and the design of its holder (**b**) [47].

For real clinical applications, it is a good choice to use surgical lamp as a light source. A simple and convenient PPGI system for contactless monitoring of anesthesia effectiveness before and during surgical procedures has been developed (Figure 12). The system involves compact lightweight camera (CMOSIS, ADC-8/10/12-bits, resolution 640 × 480 pixels, 502 frames/s, Ximea-xiQ, Cubert GmbH, Ulm, Germany) with a low-distortion lens (3.5 mm f/2.4, Edmund Optics, Barrington, NJ, USA) and green band-pass filter (half-bandwidth 520–580 nm). Both are placed in a custom designed 3D-printed case, adapted for handle attachment to the warm-white-light surgical lamp (Prismalix PRX800, ALM, Soma Technology Inc., Bloomfield, CT, USA), see Figure 12b. The camera is connected via USB-3 cable to a laptop computer which also serves as the power supply of camera. Video acquisition frame rate is 100 Hz (equal to the lamp blinking frequency), so temporal variations of the lamp intensity do not affect the PPGI signal, filtered in the frequency range 0.7–3.0 Hz. More details on the developed PPGI prototype devices and their software can be found in [42,43,44,46,47].

### 2.4. Multimodal Skin Imagers

#### 2.4.1. SkImager—A Concept Device for Multimodal Skin Imaging 

A proof-of-concept prototype device based on inexpensive components and smart software was developed for compact and handy wireless skin diagnostics and monitoring. The multimodal imaging includes capturing by a single camera a number of spectral and video-images from the skin pathology area, with subsequent extraction of clinically significant information. The device captures four consecutive imaging series: (i) RGB image of skin at white polarized LED illumination, helping to reveal hidden subcutaneous structures; (ii) four spectral images at narrowband LED illumination for mapping of the main skin chromophores; (iii) video-images under green LED illumination for mapping of skin blood perfusion; (iv) autofluorescence video-images under UV LED irradiation for mapping of the skin fluorophores. Polarized LED light is used for illumination, and round skin spot of diameter 34 mm is imaged by a CMOS sensor via cross-oriented polarizing filter. To improve the reliability of diagnostics, manipulation with maps of different parameters (i.e., extraction, summing, and division of images) is proposed, as well. Our first prototype version was described earlier [48]. 

**Figure 13:**
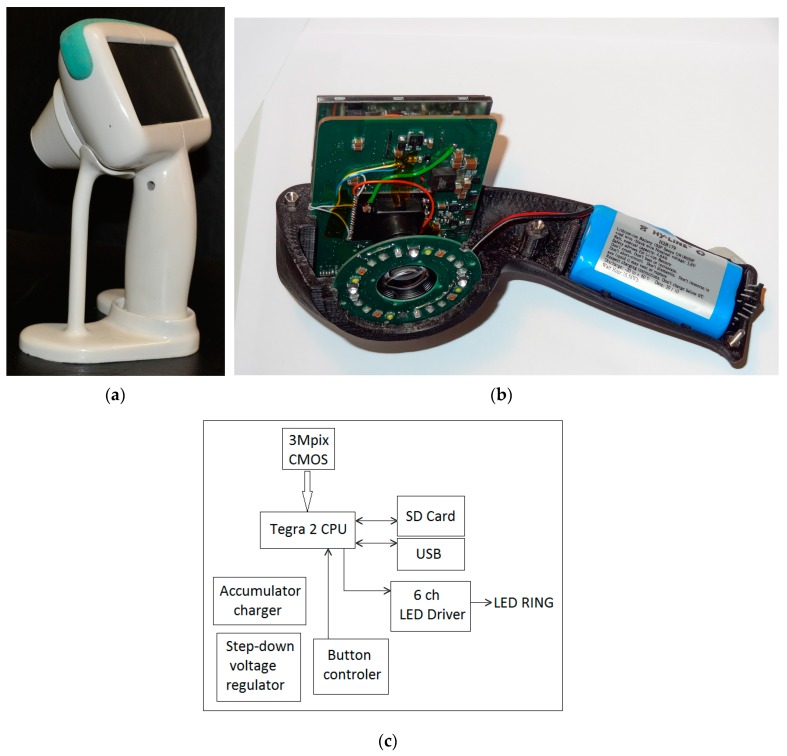
The *SkImager* prototype device in its battery-charging holder (**a**); internal design details (**b**) and functional scheme (**c**) [49].

A more advanced prototype device with the preliminary brand-name *SkImager* [49] has been developed (Figure 13). It is a battery-powered fully self-contained wireless device. Its main building blocks are CMOS image sensor, LED illumination system, on-chip microcomputer, touchscreen, memory card and rechargeable battery. The functional diagram of the device is presented at Figure 13c. A *Tegra 2 T20* (Nvidia, Santa Clara, CA, USA) system on chip (SoC) module with a dual-core *Cortex-A9* processor (clock frequency 1 GHz, ARM Inc., San Jose, CA, USA) is used as a central processing unit. It provides smooth operation of all components of the device. A 3 Mpix RGB CMOS matrix with 3.2 micron pixel size (MT9T031) serves as the image sensor; it is connected to the central processor via 10-bit parallel line. Removable SD memory card stores the image information that can be transferred to external processor, e.g., PC. It can be done also via Mini-USB connector which also ensures software installations and updates from outside. On-board and off-board calculations can be performed to extract parametric maps of the examined skin area. The main information input-output device is the built-in 4.3 inch/480 × 272 pixels touchscreen. It displays the operation mode and state of the device (battery charge level, clock, state of the memory card). Power switch button is placed on the side above the slot of memory card (Figure 13b). The device has its holder with integrated contacts for battery charging (Figure 13a). The Li-ion battery (3.6 V, 4.6 Ah) ensures up to 15 h of operation, providing about 100 full measurement cycles. The power consumption under the maximum load (display switched-on, spectral and video image recording and processing) is 3 Watts, or 1 Watt in the waiting mode. Dimensions of the device are 121 mm × 205 mm × 101 mm, weight about 440 g.

**Figure 14:**
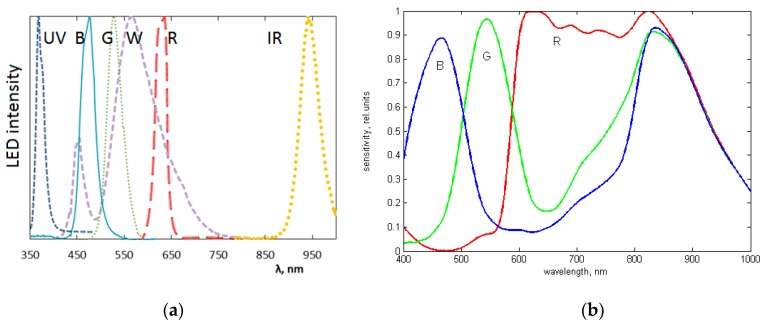
The measured emission bands of the exploited LEDs (**a**) and spectral sensitivity bands of the CMOS image sensor (**b**) exploited in the *SkImager* prototype [49].

The spectrally-specific skin illumination is performed by a ring of LEDs, surrounding the objective of image sensor—Figure 13b. In total 24 LEDs are operated—six sets of four diodes, emitting at six various wavelength bands (peaks at 365 nm, 450 nm, 540 nm, 660 nm, 940 nm and white—Figure 14a). Each set of equal LEDs is powered separately by the 6-channel LED driver (Figure 13c), in order to provide the same illumination intensity and constant signal output by the visible and NIR emitting diodes. In order to minimize detection of the surface-reflected light, crossed film polarizers cover the LED ring and camera objective (S-Mount M12 × 0.5), respectively. To keep fixed 55 mm distance to the skin, two easily changeable conical tips are used, with the target field diameters 34 mm and 11 mm, respectively. The latter is intended for more curved skin locations, e.g., on nose. Internal surfaces of the tips are black coated and multiple-step shaped, in order to suppress any side-reflected light. The RGB CMOS (Aptina Imaging, Nampa, ID, USA) image sensor with resolution of 2048 × 1536 pixels and maximal framerate 25 frames/s is used. Spectral sensitivity bands of the CMOS sensor (with removed NIR filter) are shown in Figure 14b. More details on the *SkImager* prototype and its test results are provided in [49,50].

#### 2.4.2. The Modular Multimodal Skin Imager

**Figure 15:**
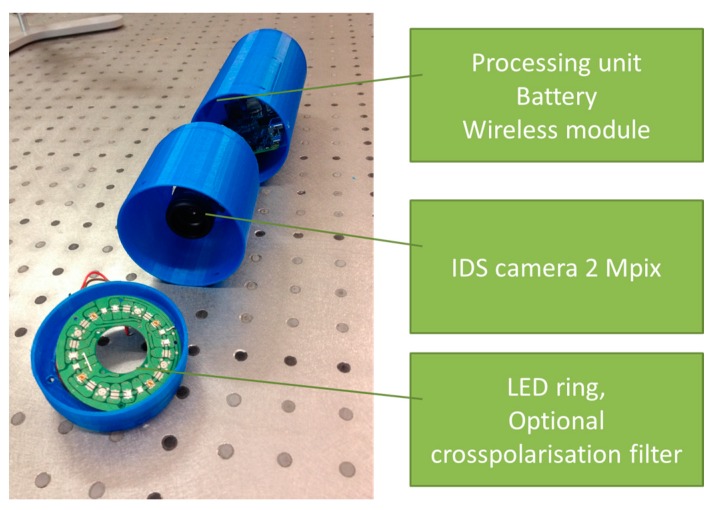
Photo of the 3D-printed modular multimodal imaging prototype [53].

Another prototype for multimodal skin imaging has been designed as a 3D-printed modular device that comprises three main modules (Figure 15):

1. Processing, wireless transmission and power block,
2. Camera and lens block,
3. Illumination block.

The first (processing and communication) module uses embedded computer—Raspberry Pi [51] as the main processing unit. Wireless connections are realized by WiPi USB dongles, and rechargeable battery is used as the power source. Charging is performed by connecting USB cable, similar as for mobile phones. All elements use standard interfaces and can be replaced just by plugging cable to the new device. The second (imaging) module holds an IDS uEye UI-3581LE-C-HQ camera [52] and the Lensagon BVM8020014 lens. Camera uses USB interface and allows upgrading to a new camera without any changes in hardware and software. IDS camera manufacturer was preferred since it has wide range of cameras and all of them share the same physical and software design. By using two lens mounts—“C type” and “S type”—lenses can be interchanged easily, as well. 

The third (illumination) module ensures three imaging modes: multispectral imaging of diffuse reflectance (spectral bands with maxima at 435 nm, 535 nm, 660 nm, 740 nm and 940 nm), fluorescence spectral imaging (excitation at 405 nm) and 635 nm laser speckle imaging. Custom printed board was created for managing skin illumination by narrowband LEDs. It uses standard interface for selecting current illumination band, therefore light source change requires less effort than in fully customized existing designs.

By using her/his own smartphone or laptop, the user connects to the device’s WiFi network and controls imaging process through the Internet browser. During the first step, the operating system’s procedure for connecting to the WiFi network (with a password-controlled access) is performed. On the step two users selects whether he/she requires a new image capture or wishes to view the previous results. During the third step user targets the skin region to be captured by viewing live video stream from the camera. Image is captured by pressing a physical button located on the device. The capturing process can be repeated a number of times. After all skin images are acquired, user can switch to the last—results viewing step. Typical time delay between finishing the image capture procedure and obtaining the results is less than 10 s. More details on this device and its tests are presented in [53].

## 3. Results

The developed skin imaging prototypes were initially tested in laboratory and then in real clinical environments. All clinical studies were performed with written consent of the involved volunteers under official approval by the local ethics committee. Some results of the clinical measurements, aimed at checking functionality and appropriateness of the prototypes, are briefly presented below.

### 3.1. Clinical Spectral Images and Chromophore Maps of Skin

**Figure 16:**
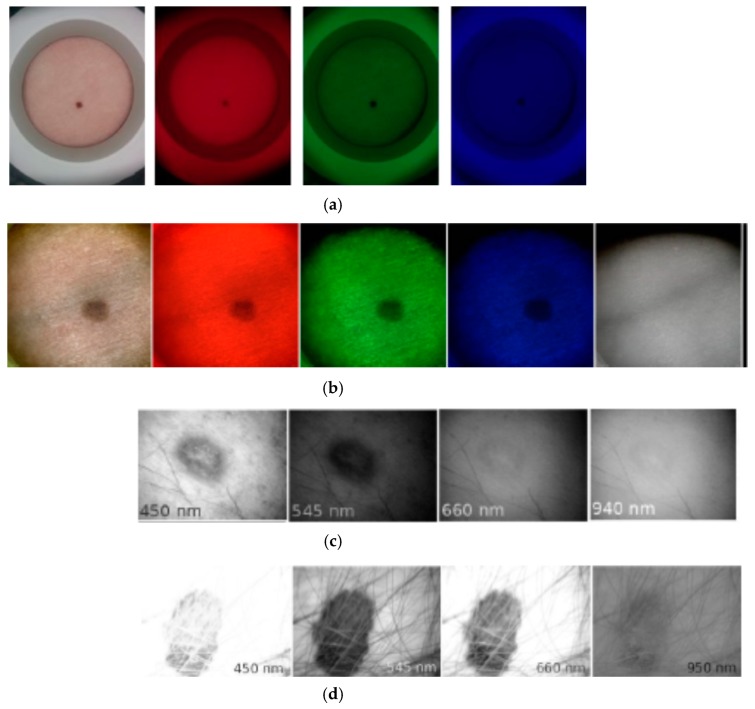
Spectral images of skin malformations taken by different prototypes: (**a**) nevus taken by smartphone—RGB LED prototype at white, red, green and blue illumination [18]; (**b**) nevus taken by SkImager at white, red, green, blue and NIR illumination [49]; (**c**) papilloma taken by modified video-microscope at blue, green, red and NIR illumination [23]; (**d**) melanoma taken by modified video-microscope at blue, green, red and NIR illumination [23].

Figure 16a illustrates spectral images of a skin nevus taken under white, red, green, and blue illumination of the RGB-LED system by *NEXUS5* smartphone camera, in comparison with spectral images of another nevus, taken under similar illumination by the *SkImager* prototype (Figure 16b). Qualitative agreement of nevi spectral images in the visible range can be observed. *SkImager* and the modified video-microscope provide also NIR spectral images at peak wavelength 940–950 nm which penetrates in skin deeper than the visible light [54]. If the NIR images of skin nevus, papilloma and melanoma are compared (Figure 16b–d), notable differences are seen. Nevus and papilloma images in NIR practically disappear while the melanoma NIR-image still has sufficient contrast, indicating to pathology in deeper skin layers accordingly to the clinical expectations [55].

**Figure 17:**
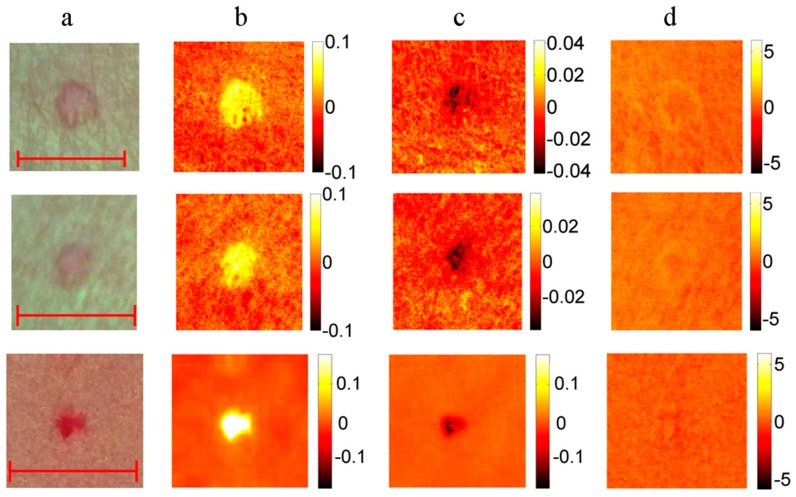
RGB image ((**a**) scale bar 5 mm) and the corresponding maps of chromophore concentration changes for three cases of a vascular hemangioma: (**b**)—oxy-hemoglobin; (**c**)—deoxy-hemoglobin; (**d**)—melanin. Units of the color scale-milimoles [28].

Nine vascular and pigmented skin lesions were examined by the mobile laser illuminator system [28]. Aim of this study was to check ability of the prototype to provide physiologically feasible skin pigmentation information on already diagnosed skin malformations. After processing the clinical images, skin chromophore maps were constructed and changes of malformation’s chromophore content with respect to the adjacent healthy skin evaluated. As initially expected, we observed notable melanin content increase in all cases of both pigmented malformations—nevi and seborrheic keratosis, without essential changes in the hemoglobin content. Increase of oxy-hemoglobin content and decrease of deoxy-hemoglobin (if compared to the surrounding healthy skin) was observed in all examined vascular malformations—hemangiomas, without notable changes in skin melanin content (Figure 17). This result is a good illustration of increased arterial blood supply in skin hemangiomas and confirms functionality of the multi-laser prototype.

### 3.2. Fluorescent and Photo-Bleaching Rate Images of Skin Tumors

Overall 50 patients with 150 different skin neoplasms were inspected with the smartphone based fluorescence imager. For more detailed image analysis 13 basal cell carcinomas (BCC) and 1 atypical nevus were selected [39]. In order to visualize the skin AF intensity decrease rates during the photo-bleaching, the following image processing expression was applied:

N(C) = (I_t0_[C] − I_t_[C])/I_t0_[C] (2)

where N(C) represents normalized AF intensity decrease map for each pixel (or pixel group) during the excitation period, I_t0_[C]—AF image at the excitation start moment, I_t_[C]—AF image after 20 s of continuous excitation and C—color component of the RGB image—red (R), green (G) and blue (B), respectively. The values of RGB components were defined from the image data by a special program developed in MATLAB^®^.

**Figure 18:**
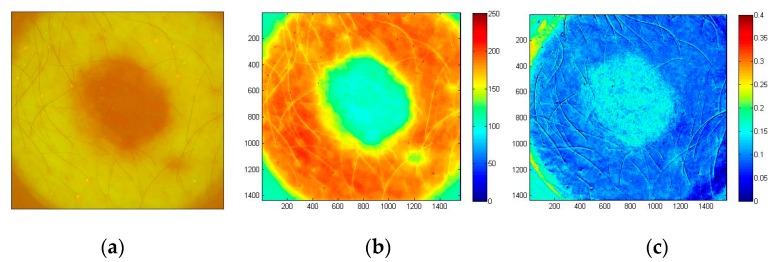
Images of solid BCC: filtered AF color image at the excitation start moment (**a**), the corresponding G-band fluorescence image (**b**) and normalized map of AF photo-bleaching rates (**c**) [39].

In all BCC cases (confirmed by cytological examination) the fluorescent images showed lowered AF intensity in malignant tissue as compared with the healthy surrounding skin, which may be attributed to lower concentration of fluorophores and increased blood perfusion caused by the malignant process. A case of solid BCC is illustrated in Figure 18. The G-band AF intensity image (Figure 18b) shows relatively low intensity within the tumor area, with clear margins between tumor and surrounding healthy skin. The tumor area also shows higher AF intensity photobleaching rate (Figure 18c) in comparison with the surrounding healthy skin. Both AF intensity and photobleaching images appear to be useful for non-contact delineation of skin tumor margins.

**Figure 19:**
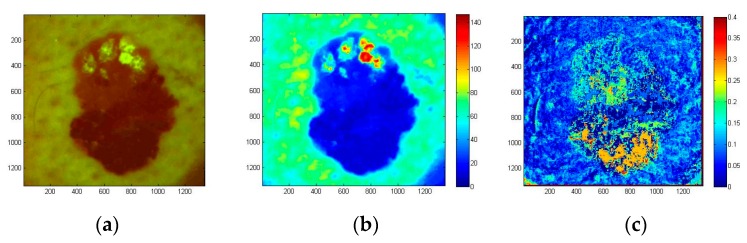
Color filtered AF image of skin atypical nevus at the excitation start moment (**a**), the corresponding G-band fluorescence image (**b**) and normalized AF photo-bleaching rates (**c**) [39].

The examined atypical nevus (Figure 19) before surgical excision was suspected as melanoma. Histological analysis of the removed tissue sample revealed three different types of tissue cells within the lesion area. Specifically, the upper part of the pathology mostly prevailed by intradermal nevus, the middle part by dysplastic nevus, and the lower part by junctional nevus. Not visible by naked eye, such triple structure of in-vivo skin malformation can be clearly seen in the image of autofluorescence photobleaching rates (Figure 19c). Normalized AF decrease distribution map before surgery/histology showed the fastest intensity decrease in the lower (junctional nevus) and upper side (intradermal nevus), while the middle part (dysplastic nevus) of lesion photo-bleached slower. This example confirms promising potential of the proposed AFPB rate imaging technique for non-contact diagnostics of oncological changes in skin.

### 3.3. Skin Blood Perfusion Measurements With the PPGI Prototypes

In order to evaluate ability of the double-wavelength PPGI prototype (Figure 10) to discriminate between cutaneous superficial and deep plexus perfusion, hyperemia of superficial plexus was induced by vasodilatory liniment (Transvasin, Seton, UK) [46]. PPGI signal amplitudes at both spectral bands of illumination (peak wavelengths 530 nm and 810 nm) were recorded; Laser Doppler imaging (LDI) signals were captured in parallel by a certified commercial device. Ten healthy volunteers (five males and five females, skin type II) were recruited to participate.

**Figure 20:**
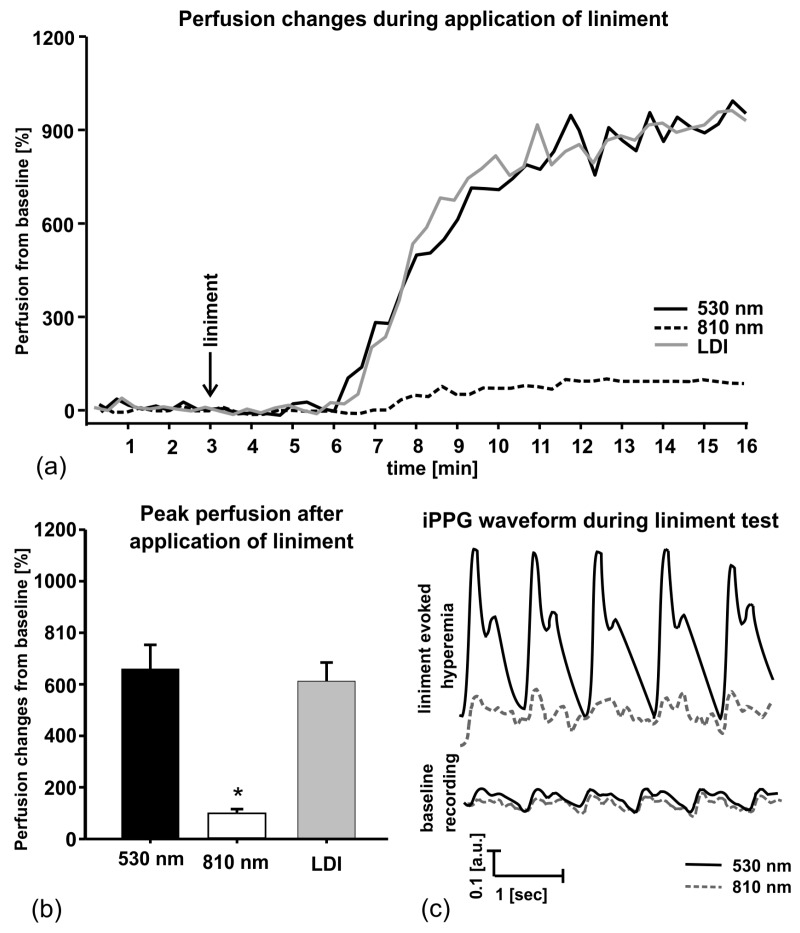
Liniment-induced skin perfusion changes as registered with the double-wavelength PPGI prototype and commercial LDI device (**a**), peak perfusion increase from the baseline (**b**) and the PPGI signal waveforms before and after provocation (**c**) [46].

The subjects were seated on a comfortable reclined chair with the right hand at the dorsal aspect, fixed on the vacuum pillow support (Figure 10b) and kept at heart level, with fingers tightly fitted to avoid movements. The protocol involved 3-min recording of the baseline followed by topical application of liniment and signal recording for 12 min. Within three to four min after application of liniment the reddening of skin appeared, indicating to hyperemia. Green PPGI measurements showed essential (about six times) increase of superficial skin perfusion, well correlated to that measured by the LDI device, while the NIR PPGI signal amplitude (reflecting arterial blood perfusion in deeper dermal layers) only slightly increased—see Figure 20. This result convincingly demonstrates the functional abilities of the developed double-wavelength prototype for non-contact skin microcirculation monitoring.

**Figure 21:**
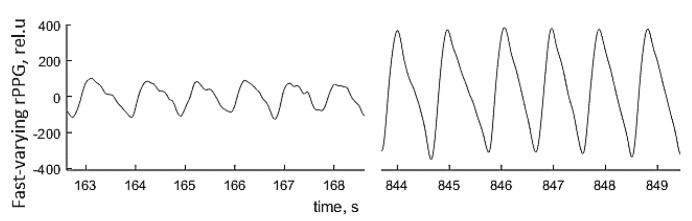
The palm PPGI signal waveforms before (**left**) and after (**right**) administration of local anesthetic [47].

Local anesthesia affects the sympathetic vascular tone, resulting in vasodilation and raised skin blood perfusion. This increases the amplitude of fast-varying signal detected by the PPGI prototypes—see Figure 21 for illustration. Such physiological response makes possible to detect remotely, if and when anesthesia works just by following the changes in amplitude of the detected PPG signals.

**Figure 22:**
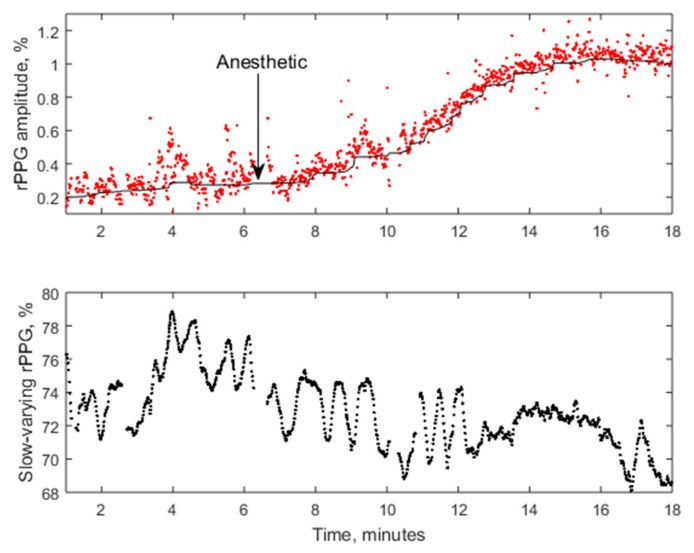
The averaged amplitude of fast-varying PPG signal (**above**) and slow-varying signal (**below**) during the regional anestesia procedure [47].

Figure 22 presents the PPG signal amplitude dynamics in response to regional anesthesia of patient’s palm before surgery, as detected by the PPGI prototype attached to the surgical lamp (Figure 12). The graph above shows the beat-to-beat amplitude dynamics while the graph below represents temporal variation of the slow-varying PPGI component. Gradual increase of perfusion with subsequent rise of the PPGI signal amplitude (the upper graph) was observed few min after the administration of local anesthetics. The plateau phase—stable increased skin perfusion induced by the anesthetic—was reached approximately 10 min later and indicated the possible surgery starting time.

**Figure 23:**
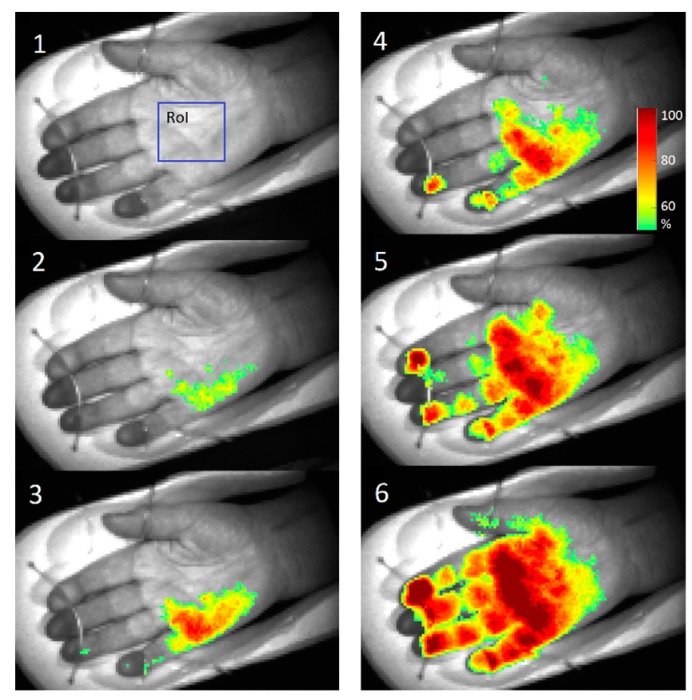
PPGA maps before (1) and after the administration of local anesthetic: 2—1 min, 3—3 min, 4—5 min, 5—6 min, 6—7 min later [47].

Figure 23 shows several screenshots of palm video fused with PPG-amplitude (PPGA) maps during anesthetic action before the surgery. As the local anesthetic affected four different nerves, subsequent microcirculation changes at four different palm skin zones (dermatomes) were observed. The PPGA maps showed increased microcirculation in dermatomes immediately after the local anesthetic was administered (stages 2–6). To conclude, the obtained results confirm clinical efficiency of the developed PPGI prototypes for remote patient anesthesia monitoring before and during surgeries.

## 4. Discussion

The principles of operation and design features of ten recently developed prototype devices for diagnostic skin imaging have been presented, along with some clinical test results confirming their practical applicability. The laboratory-made prototypes were mainly intended as proof-of-principle devices, able to examine suitability of the developed design concepts and software solutions for further implementation in real clinical environments. Diagnostic sensitivity/specificity, influence of skin color, epidermal thickness and/or optical clearing have not been considered at this stage, as they can be evaluated only after statistically representative clinical trials.

Tests of the multispectral skin imaging prototypes led to conclusions on critical conditions to be met for successful extraction of skin parametric maps (e.g., chromophore distribution maps) from the captured spectral images. The most important ones are uniformity of skin illumination at all used spectral bands, long-term repeatability of all spectral illumination parameters, possibly narrow spectral bandwidths (monochromatic spectral images preferred) and short image acquisition time (single snapshot mode preferred). The developed designs are intended for imaging of relatively flat skin areas; the curved ones (e.g., nose, fingers) still remain an issue due to uneven illumination and varying focal distance. Speckle free multi-laser illumination [33] seems to be a good challenge for multi-spectral skin diagnostic imaging in future. If smartphones or consumer cameras are used for skin image capturing, all automatic settings must be switched off to avoid unexpected artifacts. Besides hardware, also the spectral image processing software has a lot of space for improvements, in order to speed up the image conversion processes and to use more adequate algorithms for calculation of clinically significant parameters.

Fluorescent skin imaging is relatively less complicated non-contact diagnostics technique. It was shown that even a simple and inexpensive smartphone-illuminator system (Figure 9) can provide clinically interesting data for skin tumor identification and margin delineation. Imaging of skin autofluorescence photo-bleaching rates certainly has a good potential for such clinical applications. A technical challenge to be solved is ensuring motionless conditions during the recording of fluorescent images for at least 15 s from any location of the body. The camera-skin motions can be easily avoided if the skin surface is relatively flat (e.g., on the back), while obtaining high quality fluorescent video-files from curved body locations (e.g., face, arms) would require further developments of special holders and skin spacers. Like in the case of multi-spectral imaging, software development for fast and accurate fluorescent image processing is still an issue, as well.

The developed photoplethysmography imaging prototypes and software have revealed new challenges for distant non-contact monitoring of skin blood perfusion changes at different vascular depths and under anesthesia. The latter application seems to be most successful if the camera is attached to the surgical lamp or originally integrated there. Stabilized DC power supply to the lamp is preferred, but the AC supply also does not influence the results much if the video frame rate is selected equal to the light pulsation frequency. Fusion of PPGA images with real body images (Figure 22) seems to be efficient for future on-line monitoring of the anesthesia process details.

Tests of the developed multimodal imaging prototypes have confirmed their proposed functionality. However, from the point of clinical users the number of offered options seems to be too high and more specialized devices based on the tested concepts eventually might be more successful in the medical device market.

Implementation of new design ideas and practical tests of the prototypes will promote better understanding of the challenges and drawbacks of in-vivo skin imaging technologies. Their clear advantages are remote and nearly real-time operation, as well as ability of quantitative pathology documentation. On the other hand, image-based clinical criteria for skin pathology diagnostics either do not exist or are in very early stage of development. Routine healthcare needs “golden standards” for fair assessment of patient’s condition and for setting up the recovery strategy, and from this point optical imaging techniques still have to be checked at numerous clinical measurements before becoming a standard of care. Even if some of the above-described skin imaging prototypes would appear clinically acceptable, lots of efforts will be needed in future to establish and validate specific image parameters related to clinically significant threshold levels of particular skin pathologies.
